# Remarks on the Obstetrical Forceps, with a Description of an Instrument Employed

**Published:** 1850-01

**Authors:** 


### ARTICLE III.

*Remarks on the Obstetrical Forceps, with a Description of an Instrument Employed.*—By JAMES P. WHITE, M.D., Prof. of Obs., etc., in the Med. Department of the University of Buffalo. (From the May No. of the Buffalo Med. Journal.) From the Author.

This is a small pamphlet of eight pages, with a cut, that has been on our table for some time; and but for misplacement, would have received earlier attention.

After a brief review of the forceps in Europe, with an account of the forms of the instrument most in vogue, the author says of the instrument as used in this country—"Most of those which I have examined are bungling modifications of Dewees' improvement of Baudeloque's or of Seibolds."

The author thinks Dr. Hodge's "*Eclectic Forceps*" the best in use, but points out the defect, which we have found to be a serious one, which is the want of sufficient strength in the instrument to prevent it from giving so as to slip off of the head of the child.

The instrument which the author modestly says has been used by himself, is a beautiful one, and evinces the author's ingenuity, as it was not only used, but planned by him. We had the pleasure of examining Prof. White's forceps last spring, and if he has not carried the idea of extreme attenuation and lightness too far, we doubt not it will prove to be one of the best patterns yet devised. It is lighter than Prof. Hodge's, but the author says on account of the angle in Prof. Hodge's instrument, designed to adapt the blades to the head so as not to distend the maternal passage in advance of the presenting part too much, his will bear much greater force than they will without springing. Our opinion, however, is, that if Prof. Hodge's instrument were made of sufficient strength to prevent it from slipping off by the elasticity of the handles, the angle that adapts the blades closely to the head, so as to prevent the distension of the maternal passage in advance of the head, would be a valuable improvement, which our author has sacrificed to the idea of making the lightest, instrument.

We are at a loss to know why a sufficient amount of metal may not with propriety be put in the forceps, to render them strong enough, with any shape that might be most desirable, to answer the end for which they are designed.

There is an ingenious arrangement in one of the handles of Prof. White's forceps, by which the blunt hook is taken off, leaving the tenon to form a perforator, so that the instrument may serve entire as forceps—one blade as a vectis—the handle as a blunt hook, and also as a perforator. The handles are roughened to prevent the hand from slipping, which is certainly a good arrangement. E.

